# Linear programing formulation of a high temporal and technological resolution integrated energy system model for the energy transition

**DOI:** 10.1016/j.mex.2022.101732

**Published:** 2022-05-16

**Authors:** Manuel Sánchez Diéguez, Amirhossein Fattahi, Jos Sijm, Germán Morales España, André Faaij

**Affiliations:** a University of Groningen, Groningen, Netherlands; b TNO Energy Transition, Netherlands

**Keywords:** Demand response, Demand-side management, Flexibility, Demand shedding, Smart charging, Vehicle-to-grid, Infrastructure

## Abstract

Models with a wide technological representation of energy systems can hardly adopt hourly resolutions to study the energy transition towards low-carbon technologies due to extended problem size. This compromises the model's ability to address the challenges of variable renewable energy sources and the cost-effectiveness of cross-sectoral flexibility options. This methodology presents a linear program model formulation that simultaneously adopts different temporal representations for different parts of the problem to overcome this issue. For instance, all electricity activities and their infrastructure representation require hourly constraints to better replicate system feasibility. The operation of gaseous networks is settled out with daily constraints. The balancing of the other activities of the system is represented with yearly constraints. Furthermore, the methodology adopts an hourly formulation to represent in detail 6 cross-sectoral flexibility archetypes: heat and power cogeneration, demand shedding, demand response, storage, smart charging and electric vehicles. The model can successfully solve the transition problem from 2020 to 2050 in 5-year intervals with more than 700 technologies and 140 activities (including the electricity dispatch of the Netherlands and 20 European nodes) in less than 6 hours with a normal computer.

• Different temporal scales for the representation of different activities in the energy system.

• A high-resolution hourly description for the formulation of cross-sectoral flexibility in integrated energy models.

## Graphical Abstract

**Figure:**
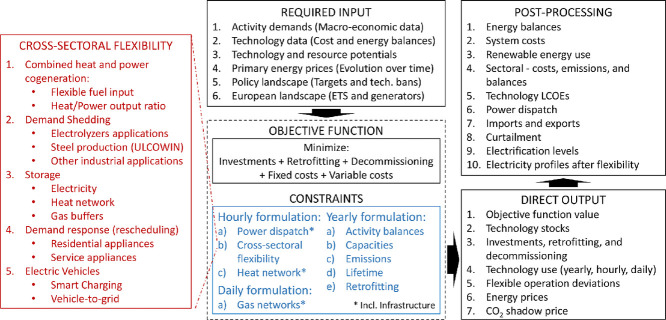

## Nomenclature

**Indices**
p: Index of the set conformed by all the modelled periods
h: Index of the set conformed by all the hours in a year
d: Index of the set conformed by all the days in a year
a: Index of the activities set
ae: Index of the electricity-related activities subset, $A^{e}$
ah: Index of the national heat-related activities subset, $A^{h}$
ag: Index of the gas-related activities subset, $A^{g}$
$t,t_{i},\;t_{j}$: Indices of the technologies set
te: Index of the technologies representing air released emissions in the considered target scope.
td: Index of the dispatchable technologies subset
tp: Index of the operation technologies subset
tf: Index of the flexible technologies subset
$tf_{b}$: Index of the flexible technologies of the battery type subset
tc: Index of the flexible CHP technologies subset
ts: Index of the shedding technologies subset
ti: Index of the infrastructure technologies subset

**Parameters**
$VC_{t,p}$: The variable cost of technology in a period
$\alpha_{t}$: Annuity factor of a technology (or, in this case, the inverse)
$IC_{t,p}$: Investment cost of technology in a period
$DF_{t}$: Fraction of the capital cost of a technology that remains after premature decommissioning
$RC_{t_{i},t_{j},p}$: Retrofitting costs from one technology to another
$FC_{t,p}$: The fixed operational cost of technology in a period
$AB_{t,a,p}$: Activity balance of inputs and outputs of a technology
$V_{a,p}$: Exogenous required activity volumes in a period
$\Gamma_{t}$: Available use of a technology per unit of capacity
$E_{p}$: Absolute CO_2_ emission target in a certain period.
$RM_{t_{i},t_{j}}$: Binary matrix specifying which technologies can be retrofitted into others
${S^{min}}_{t,p},\;{S^{max}}_{t,p}$: Minimum and maximum allowed installed capacities of technology in a year
$P_{h,tp}$: Hourly availability or reference operational profile of a technology
$AE_{t,a}$: Binary parameter indicating the hourly electricity activities of a technology
$R_{td,p}^{dw},\;R_{td,p}^{up}$: Ramping up and down limits of hourly dispatchable technologies
$\eta_{tc}$: Only heat reference efficiency of a flexible CHP
$\varepsilon_{tc}$: Only power reference efficiency of a flexible CHP
$SC_{ts}$: Power shedding of a technology per unit of capacity
$UtP_{ts,p}$: Use-to-power ratio of a shedding technology in a period
$SF_{ts}$: Maximum allowed shedding fraction of a shedding technology
$AG_{tf,a}$: Binary parameter indicating the gas activities of a technology
$FC_{tf}$: Flexibility capacity in terms of the impact on the corresponding network of technology.
$NN_{tf}$: Non-negotiable load of flexible technologies.
$CC_{tf}$: Charging (or discharging) capacity of a storage technology.
$CT_{tf}$: Charging time of a storage technology.
$VU_{tf}$: Hourly profile of the usage of a flexible vehicle (not connected to the grid).
$AS_{tf}$: Average speed of a flexible vehicle.

**Variables**
Symbol: Description
$u_{t,p}$: Use of technology in a period
$i_{t,p}$: Investments in technology in a period
${d^{pre}}_{t,p}$: Premature decommissioning of a technology in a period
$r_{t^{i},t^{j},p}$: Retrofitting from one technology to another in a period
$s_{t,p}$: Stock (installed capacity) of a technology in a period
${d^{cum}}_{t,p}$: Cumulative decommissioning of a technology in a period
${d^{lt}}_{t,p}$: Decommissioning of a technology in a period due to lifetime expiry
$u_{h,td,p}$: Hourly use of a dispatchable technology in a period
$\Delta {q^{up}}_{h,tf,p}$: Increase in electricity demand from a flexible technology in an hour in a period
$\Delta {q^{dw}}_{h,tf,p}$: Decrease in electricity demand from a flexible technology in an hour in a period
$\Delta u_{h,tc,p}$: Deviation in use of a flexible CHP technology in an hour in a period
$\Delta p_{h,tc,p}$: Deviation in power output of a CHP technology in an hour in a period
$\Delta u_{h,ts,p}$: Decrease in use of a shedding technology in an hour in a period
$l_{h,tf,p}$: Losses from deviations in use of flexible technologies in an hour in a period
$\Delta {q^{max}}_{h,tf,p}$: Maximum increase limit of power demand of a flexible technology in an hour
$\Delta {q^{min}}_{h,tf,p}$: Maximum decrease limit of power demand of a flexible technology in an hour
${v^{max}}_{h,tf,p}$: Upper saturation limit from shifted volume in an hour in a period
${v^{min}}_{h,tf,p}$: Lower saturation limit from shifted volume in an hour in a period
$u_{d,td,p}$: Daily use of a dispatchable technology in a period
$\Delta {q^{up}}_{d,tg,p}$: Upwards deviation in the use of a daily storage technology in a period
$\Delta {q^{dw}}_{d,tg,p}$: Downwards deviation in the use of a daily storage technology in a period

**List of abbreviations**
CCUS: Carbon Capture Utilisation and Storage
CHP: Combined Heat and Power
CO_2_: Carbon dioxide
DSM: Demand Side Management
ESOMs: Energy System Optimization Models
ETS: Emission Trading Scheme
EU: European Union
EV: Electric Vehicle
GHG: Greenhouse Gas
GTS: Gasunie transport service
HD pipeline: High-Density pipeline
HV grid: High Voltage grid
IEM: Integrated energy models
LD pipeline: Low-Density pipeline
LV grid: Low Voltage grid
LP: Linear programming
LULUCF: Land Use, Land-Use Change, and Forestry
MACC: Marginal Abatement Cost Curve
MD pipeline: Medium Density pipeline
MV grid: Medium Voltage grid
MILP: Mix-integer linear programming
PBL: The Netherlands Environmental Assessment Agency
P-to-X: Conversion of electricity (power) to a different energy carrier or product (e.g., hydrogen or ammonia)
TNO: Netherlands Organization for Applied Scientific Research
V-to-G, V2G: Vehicle to Grid
VRES: Variable renewable energy sources

## Direct Submission or Co‐Submission

“Modelling of decarbonisation transition in national integrated energy system with hourly operational resolution” [1]

"Measuring accuracy and computational capacity trade-offs in an hourly integrated energy system model" [2]

SPECIFICATIONS TABLE

**Table:** 

Subject Area;	Energy
More specific subject area;	*Integrated energy system modelling*
Method name;	*Optimisation energy system model*
Name and reference of original method;	*Integrated energy system analysis (IESA-Framework.*[3])
Resource availability;	*Website of the IESA-Opt mo*del (https://energy.nl/iesa/)

## Introduction

Energy system optimisation models (ESOMs) are a tool that allows us to identify ways of reaching decarbonisation targets in a cost-optimal way. To do so, they model the operation of the technologies present in all the system sectors using and producing energy and emitting CO_2_ and the investments behind those technologies for the entire energy transition period. Due to this scope, IEM presents an extensive versatility and can be used for many different purposes, such as exploring technology configurations, providing policy advice, and analysing development paths. The suitability of ESOMs for different applications depends on the granularity and detailing of the model. For instance, a crucial topic for the energy transition is to analyse the role that variable renewable energy sources (VRESs) play in different sectors of the energy system. However, to adequately address the topic, it is necessary to correctly account, at different points of the transition, for the hourly operation of VRES and flexible sectoral technologies able to help with the challenges brought by VRES [4]. The latter presents a significant computational challenge due to the large problem size resulting from the high sectoral, technological, spatial, and temporal resolutions required, resulting in the need for modelling choices to address the issue. To understand this, different key modelling elements must be considered, notably the ones enlisted below:

• The whole transitional period from 2020 to 2050 with perfect foresight;
• Hourly sequential representation in the operation of technologies connected to the electricity network;
• All the sectors of the energy system modelled simultaneously, accounting for crucial feedback;
• Consideration of all of the GHG emission sources that are accounted for within reduction targets;
• A wide representation of the different technologies able to provide flexibility to the system, acknowledging their operational constraints;
• An adequate temporal resolution for technologies connected to gaseous networks (hydrogen and natural gas);
• A representation of the key infrastructure networks enabling the transport of energy carriers in the system.

ESOMs have been used extensively in the energy modelling community; however, they come with their own shortcomings, and a model that considers all the above elements simultaneously does not exist. The TIMES model [5], for example, provides a detailed techno-economic representation of all energy sectors, sector coupling technologies, and infrastructural limitations while using “integral”^1^ time slices instead of hourly temporal resolution. TIMES can allow for hourly modelling instead of time slices, but due to the impracticality of the resulting problem size, it cannot be found in academic publications. Using aggregated time slices overestimates the potential contribution of large base-load power plants and underestimates the need for supply-demand management and storage with high shares of VRES [6]. Like TIMES, OPERA provides a detailed techno-economic representation of the energy system; however, it lacks the multiperiod optimisation methodology [7]. Neglecting the multiperiod optimisation undervalues the role of the current technological stock and its techno-economic lifetime on system costs. PyPSA provides an open-access energy system model that emphasises power network details such as the physics of power flow according to the impedances in the network [8] at the expense of a simpler technological representation of other sectors. Compared to other ESOMs, OseMOSYS requires less time commitment to operation, and being open-source, it requires no upfront financial investment; however, it lacks the inclusion of high technological details and infrastructure constraints [9]. REMix uses the EnDAT tool [10] to preprocess the heat and power demand data for incorporating geospatial variations in the hourly optimisation model [11]. However, the model's main focus is the power system and does not provide a complete sectoral description of the energy system and its emissions. A model presented by Göke in 2021 addresses the energy transition while allowing for different spatial and temporal resolutions for different energy carriers [12], but it uses aggregated volumes to identify the energy carriers demand projections rather than base them on economic activities. Many other models also address the above elements but were omitted from this brief literature review to avoid redundancies. However, a complete literature review^2^ was carried out before, in which an extensive list of models was explored [3]. None of the models identified addressed all the elements simultaneously.

To fill this knowledge gap and to be able to provide a complete and comprehensive approach to study low-carbon potential scenarios with high levels of VRES for the transition in the Netherlands, we developed an integrated energy system model named IESA-Opt. This model has already been used to explore the decarbonization transition in the Netherlands’ energy system [1], and to measure the results impact and computational weight of modelling capabilities [2]. IESA-Opt is an optimisation model using a linear programming (LP) formulation to determine the cost-optimal investment path in the transition towards 2050 decarbonisation targets and the operation of the technologies present in the system. An LP approach allows for representing the energy system with high sectoral, technological and temporal resolution while maintaining computational feasibility^3^. The chosen formulation also allows for the flexible framework used in the model, which enables the energy system to be described in clusters or to include geographical constraints of the model^4^. Conventional large-scale, long-term planning energy system models frequently use LP methodology to avoid excessive computational loads. Due to their narrower system scope, operational energy system models, especially power system models, employ a mixed-integer linear programming (MILP) methodology to account for binary or integer variables such as investment and unit-commitment decisions. The choice of LP over MILP methodology can considerably reduce the computational time without important deviations in the results, especially in energy systems with high shares of VRES [13]. The computational time of the LP formulation can be significantly lower than that of the MILP approach (up to 100 times) while providing relatively high precision in modelling relevant flexibility options [14]. The most significant modelling sacrifice of not using an MILP approach is that the concept of economies of scale cannot be represented through convex functions. However, the latter downside is counterweighted by the higher resolution of the activities considered by the model, which allows for different policy guiding approaches. Unfortunately, adequate testing of this hypothesis would require a contrasting MILP formulation that cannot be feasibly solved for such a large problem at reasonable times without the need for supercomputers.

## IESA‐Opt conceptual framework

**Fig. 1:**
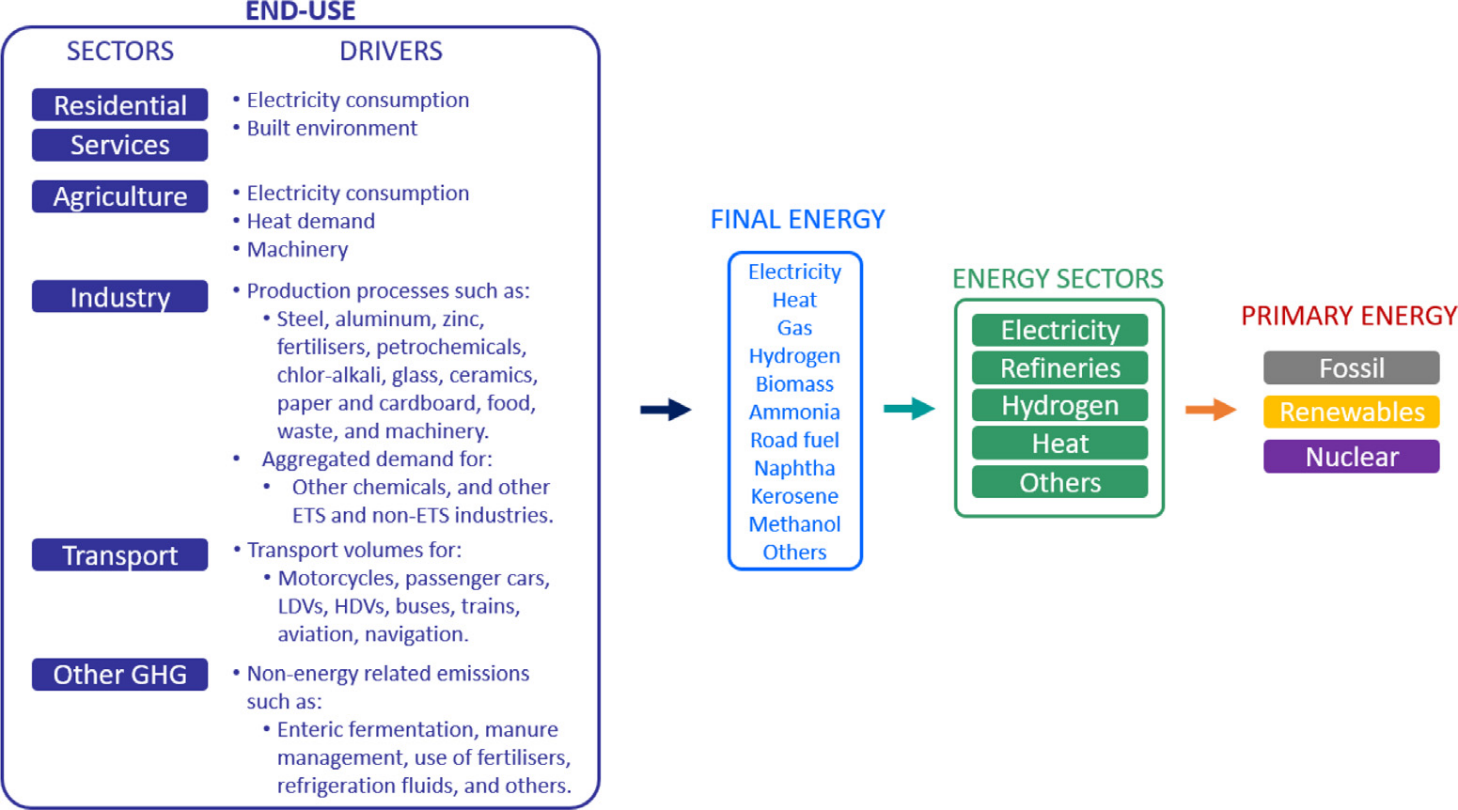
IESA-Opt conceptual framework.

To include all the activities of the energy system, the model differentiates between driver activities and energy activities. Being the driver activities those who create the need to use energy in the first place (e.g., the production of steel or the use of passenger cars), and the energy activities corresponding to specific forms of energy carriers (e.g., electricity or hydrogen). This means that the model needs to be fed (exogenously) with the projected production (or usage or demand) volumes of the driver activities, data often found in macroeconomic projections. However, it endogenously determines which technologies are used to meet such volumes accordingly with the ‘menu’ of technological options presented to the model. Such a menu of options requires cost data and efficiency data to describe a technology, making technology learning a key model input. Simultaneously, the presence and operation of the aforementioned technologies create the need for energy in diverse forms, for which the model determines (also endogenously) the technological choice, installed capacities, and operation to supply them (also based on the inputted ‘menu’ of technological options). It is important to mention that the extent to which the system can adopt a technology is constrained by an assumed potential, making those potentials a key element of a scenario description. Finally, it is the operation of technologies to satisfy both driver and energy activities that generate emissions and the demand for primary energies, completing the required remaining panorama to determine the cost-optimal system configuration. A visualisation of the previously described conceptual framework is presented in Figure 1.

As mentioned before, to provide cost-optimal planning towards complete system decarbonisation, IESA-Opt adopts very high sectoral, technological, and temporal granularities. This means that all the important energy-consuming activities are described in the model and that a large variety of technology options are considered to satisfy them. First, to explore cross-sectoral feedbacks (and coupling), it presents a sectoral bottom-up representation of standard and “low-carbon” options comprising biomass, CCUS, electrification and VRES, which result in a detailed description of considered activities and technologies. Then, the model considers hourly intrayear resolution, adequate to cope with the challenge presented by the adoption VRES [15]. Additionally, the latter requires that the model includes features that enable it to explore the roles that interconnected European power markets and flexibility alternatives play to further adopt VRES [16, 17]. Finally, the model also provides infrastructure descriptions such as pipelines and buffers for natural gas (LD, MD, HD), hydrogen (LD, HD), CCUS, and district heating and transmission lines and transformers for electricity networks (North Sea, LV, MV, HV). These descriptions help to account for costs and potentials to feasibly integrate VRES via their coupling with other energy carriers into the system, such as gas, hydrogen, or heat, and their possible synergies with CCUS [18,19].

The linear formulation behind the representation of the above conceptualisation is presented in the following sections. Section 3 presents how to simultaneously integrate the operation of all sectors, activities, technologies, and emissions under one model and one objective function. Section 4 presents the formulation representing the evolution of technological stocks resulting from investment, decommissioning, and retrofitting decisions. The LP representation of the power dispatch (a key element of the energy system) is described in Section 5. Next, the formulation behind the flexible operation of technologies is presented in Section 6, where the most meaningful methodological contributions of the paper can be found. Section 7 describes the operational constraints of gaseous networks. Finally, the representation of networks’ infrastructure in the energy system can be presented in Section 8. All the model resources can be found at https://energy.nl/iesa/.

## Sectoral integrated cost‐optimised energy system towards decarbonisation targets

As described in the above presented conceptual framework, sectoral integration in IESA-Opt turns around two main axes, activities and technologies (analogous to the commodities and processes nomenclature in TIMES [20]). Thus, many technology use combinations can satisfy a desired volume of activities under a richly described technological landscape. The model simultaneously determines the optimal configuration and use of technologies to satisfy the required activities’ volumes from such a broad domain. It minimises system costs resulting from the set of decision variables confirmed by use, investments, decommissioning, and retrofitting of technologies accordingly with the following expression^5^.

(1) $$\min\left[\sum_{t,p}u_{t,p}VC_{t,p}+i_{t,p}\alpha_{t}IC_{t,p}+{d^{pre}}_{t,p}DF_{t}\alpha_{t}IC_{t,p}+r_{t_{i},t_{j},p}\alpha_{t_{j}}RC_{t_{i},t_{j},p}+s_{t,p}FC_{t,p}\right]$$

Subject to ensure that the use of technologies meets at least the required exogenous activities drivers, as described by

(2) $$\sum_{t}u_{t,p}AB_{t,a,p}\geq V_{a,p}$$

Additionally, subject to the available installed capacities of the technologies and the particular activity-to-capacity ratio for each technology, as shown in (3), $\Gamma_{t}$.

(3) $$u_{t,p}\leq s_{t,p}\Gamma_{t}$$

Every single technology can affect one of the following emission-related activities considered in the model: CCUS network, national ETS, national non-ETS, external ETS, and international transport emissions. Most technologies increase the net volume of the emitting activity, and some technologies decrease it (such as carbon capture and direct air capture). To keep the emission activities balanced, four ‘technologies’ match their net account: CO_2_ released to air in the national ETS, national non-ETS, external ETS, and international transport accounts. The emission constraint is therefore enforced by ensuring that the CO_2_ released to air in the national ETS and non-ETS accounts does not exceed the national targets defined for the different periods as described by the following constraint:

(4) $$\sum_{te}u_{te,p}\leq E_{p}$$

Nevertheless, it is important to mention that not all the sources of emissions considered within the scope of the targets are included within the activities covered by IESA-Opt. To be precise, approximately 85% of the emissions considered within the 2021 national inventory [21] are covered by the activities included in the energy system framework; then, for the remaining 15% (mostly agricultural activities), a less detailed approach is used. Here, the emissions resulting from activities such as enteric fermentation, manure management, use of fertilisers and use of refrigeration fluids are input to the model as driving activities. Their potential reductions and costs are addressed with MACC curves (extracted from the IMAGE model database [22]). A complete description of the methodology is provided in Appendix B **Error! Reference source not found.**.

## Transition path

The transitional capability of the model derives from the fact that it can plan for the optimal system configuration for the different periods covered in the transition, at the same time that it determines the optimal intra-year operation of the stocks. The transitional elements are described by the investment, premature decommissioning, and retrofitting decisions that give shape to the technological stock accordingly with the following formulation:

(5) $$s_{t,p}=s_{t,p-1}+i_{t,p}+r_{t^{i},t,p}-r_{t,t^{i},p}-\left({d^{cum}}_{t,p}-{d^{cum}}_{t,p-1}\right)$$

being:

(6) $${d^{cum}}_{t,p}={d^{cum}}_{t,p-1}+{d^{pre}}_{t,p}+{d^{lt}}_{t,p}$$

It is important to ensure that premature decommissioning can freely happen at any convenient period but avoid decommissioned technologies that cannot be decommissioned in a year and recommissioned back in a subsequent period. Simultaneously, the model must be able to address the costs of premature decommissioning. For this purpose, the following constraint together with (5) and (6) ensures that both requirements are satisfied:

(7) $${d^{cum}}_{t,p}\geq {d^{cum}}_{t,p-1}$$

Additionally, as part of the scenario descriptions, some technologies are defined within a certain deployment bandwidth. This same constraint, depicted in (8), sets the adoption potentials for technologies and caps system emissions.

(8) $${S^{min}}_{t,p}\leq s_{t,p}\leq {S^{max}}_{t,p}$$

Last, the retrofitting of technologies is constrained by the available stocks of the original technology and by an input binary parameter that determines which are the possible retrofitting relations. This results in the following formulation:

(9) $$r_{t_{i},t_{j},p}\leq s_{t,p-1}RM_{t_{i},t_{j}}$$

## European hourly power sector dispatch

Modelling power dispatch within ESOMs asks for choices to be made to avoid enormous computational requirements. First, the study [23] concluded that poor temporal resolutions negatively affect outcome reliability for scenarios with moderate and high presence of VRES and greatly recommends prioritising using at least hourly resolution. Additionally, adopting a sequential description of the power dispatch enables us to retain the chronological order in the variability of the events, which is key for short- and long-term storage technologies. Thus, IESA-Opt adopted an hourly resolution of the complete year operation (8760 sequential points per year).

Furthermore, the same study [23] also mentions that operational detailing, namely, unit commitment, increases reliability as the presence of VRES starts to increase. However, it also states that adopting unit commitment loses relevance after a certain level of VRES penetration, as fewer thermal units affect the system dynamics. This observation is further reinforced by another study that states that MIP unit commitment performs better in scenarios with a low presence of VRES, but for scenarios with high levels of VRES, an LP approach suffices to provide reliable results [13]. Additionally, there is plenty of evidence that increasing the geographical scope of the model to consider European cross-border interactions has a significant impact on the outcome reliability of the models [24,25]. Therefore, in this model, we exclude the unit commitment formulation (MIP) and rather include the whole European power system represented in 20 nodes (see Appendix C). This penalises the ability of the model to reliably analyse low VRES scenarios with a high presence of thermal generators (as unit commitment is excluded), but keeping the convenient LP formulation enables IESA-Opt to simultaneously solve the EU power dispatch and the integrated national energy system within the same formulation while considering a high temporal resolution and a moderate and high presence of VRES. Thanks to such modelling choice, it is possible to analyse the interaction of storage, flexible demand technologies, VRES, and cross-border interconnection within the sector-coupled energy system of the Netherlands.

The following linear formulation is used to include the previously described concepts within the IESA-Opt framework. First, the fundamental constraint that the electricity supply and demand must remain balanced every hour is included. For this purpose, we divide technologies into five main groups: dispatching technologies, $t_{d}$, technologies with flexible, $t_{pf}$, and nonflexible operation, $t_{pn}$, flexible CHPs, $t_{c}$, and shedders, $t_{s}$. For each of the 24 different electricity networks considered in the model, conforming to the set $A^{e}$, the hourly balance is represented with the following constraint:

(10) $$\begin{array}{ccc} u_{h,td,p}AP_{td,a,p} & = & u_{t_{p},p}P_{h,tp}AB_{tp,a,p}+\left(\Delta {q^{up}}_{h,tf,p}+\Delta {q^{dw}}_{h,tf,p}\right)AE_{tf,a}\\ & & +\;\left(u_{tc,p}P_{h,tc}+\Delta u_{h,tc,p}\right)AB_{tc,a,p}+\Delta p_{h,tc,p}AE_{tc,a}\\ & & +\;\left(u_{ts,p}P_{h,ts}+\Delta u_{h,ts,p}\right)AB_{ts,a,p}\;\forall \;a\;a\;\in \;A^{e} \end{array}$$

This equation can be read as supply is equal to reference hourly demand, plus flexible demand variations ($\Delta {q^{up}}_{h,tf,p}$ and $\Delta {q^{dw}}_{h,tf,p}$), plus the bidimensional CHP flexibility variations ($\Delta u_{h,tc,p}$ and $\Delta p_{h,t_{c},p}$), and the shedding demand variations ($\Delta u_{h,ts,p})$, for each interconnected node. These three forms of flexibility are further explained in section 6.

Another major determinant for the dispatch of electricity is resource availability, and this turns relevant for two reasons: the installed capacities of generation technologies and the intermittency of renewable energy sources. Every technology in the model is described with an hourly operation $P_{h,t}$. For the dispatching technologies, this profile represents the hourly availability of the resource, and for the other technologies, it represents the hourly reference operation^6^. The availability of VRES resources can substantially impact the energy system outcome [26]; hence, the ability of the model to easily modify the profile of any technology in the system is a significant characteristic. The following constraint ensures that supply occurs according to the existing installed capacity and to the extent to which hourly resource availability allows it:

(11) $$u_{h,td,p}\leq s_{td,p}\Gamma_{td}P_{h,td}$$

Additionally, ramping constraints are considered for dispatchable generation according to the following constraint:

(12) $$-R_{td,p}^{dw}\leq \left(u_{h,td,p}-u_{h-1,td,p}\right)\leq R_{td,p}^{up}$$

Losses occurring during the transport process are accounted for only when energy is “transferred” from one network to another by a capable technology (connector, transformer, compressor). Hence, the formulation does not account for losses proportionally to travelled distance under a specific voltage level and cable type. The formulation of the considered losses is implicitly modelled in the energy balance of the technology and therefore driven by the use of such technology.

Last, the European representation, the dispatch architecture, the data on profiles and operational parameters are strongly based on the same modelling structure used as input by the COMPETES model [27]. Further details can be found in Appendix C.

## Hourly flexible operation in coupled sectors

In addition to the power dispatch description, representing possible deviations from reference hourly operation profiles is paramount for the dispatch and adequately represents sector coupling. With this aim, IESA-Opt considers three types of intrayear operational decisions: flexible CHPs, shedding technologies, and demand technologies with flexible operation.

### Flexible CHP's

CHPs are modelled as operation technologies, which means that their hourly operation profile is fixed, and the changes in their use affect such profiles proportionally. However, some CHPs, known as extraction-condensing steam turbines, can extract a fraction of the condensed steam before (or during) the expansion phase (the power turbine) to be used to provide heat [28,29]. Such enhancement allows these turbines to adjust their power-to-heat ratio, which, combined with the amount of steam generated before the expansion, gives the technology a huge potential to modify its power and heat outputs and fuel inputs to adapt to electricity price events (among other externalities) [30]. The resulting bidimensional flexibility (the fuel inputted into the boiler and the extraction flow of the condensed steam) is considered by IESA-Opt using a convenient LP simplification (resembling other ESMs [31]).

In a linear representation of a flexible CHP, the fuel requirement, F, is assumed to be determined by the heat and power outputs, H and P, accordingly with F=H/η+P/ε. where η and ε represent the CHP efficiencies when producing only heat and power, respectively. For this, IESA-Opt considers two dimensions of flexibility: the hourly deviations in the fuel input representing the deviations in use, $\Delta u_{h,t_{c},p}$, and the hourly deviations in the power output, $\Delta p_{h,t_{c},p}$. This leads to the following constraint to ensure that the heat demand provided by the CHP is satisfied in a specific time window:

(13) $$\sum_{h\;\in \;TW_{tc}}\left[\left(u_{tc,p}P_{h,tc}+\Delta u_{h,tc,p}\right)AB_{tc,a,p}-\eta_{tc}/\varepsilon_{tc}\;\Delta p_{h,tc,p}\right]=\sum_{h\;\in \;TW_{tc}}u_{tc,p}P_{h,tc}AB_{tc,a,p}$$

### Shedding technologies

The upcoming energy transition will deliver a set of technologies that could provide sector coupling via the conversion of electricity into other energy forms (such as heat [32], hydrogen [33], methanol [34], methane [35], hydrocarbons [36], chlorine [37], ammonia [38], and other chemicals [39]) via technologies such as heat pumps or electrolysers. Additionally, some industrial processes (such as electrified steel production, aluminium smelters, and paper pulp mills) can stop or lower their activity level to adapt to power market dynamics. We use word shedding to refer to the action taken by all of the abovementioned technologies of cutting down operations in a critical hour to decrease electricity consumption and help to alleviate the system. This opens the door to foreseeable scenarios where these technologies could be interruptedly operated to avoid high electricity price events and decrease operational costs [39]. However, extra capacity must be installed to satisfy demand while sacrificing operational times [40]. In summary, shedding technologies in IESA-Opt can selectively operate in specific hours in exchange for overinvestments.

The representation of these technologies in the model assumes they can shed their hourly activities using an hourly decision variable that represents the decrease in use for each hour. This variable is capped by the installed capacity of the technology, as shown below:

(14) $$\Delta u_{h,ts,p}\leq s_{ts,p}SC_{ts}UtP_{ts,p}$$

Because, as stated in (2), the model must ensure sufficiency in the activities balances, it will determine the required technological stock, determining the necessary excess capacity to cope with such shedding.

Furthermore, technologies might not have a flat operational profile and might be subject to specific sectoral dynamics, or perhaps a certain technology may require a minimum level of operation, such as heat pumps with seasonal heat storage or P-to-X in industry. For these cases, shedding will occur between the reference operational profile and the minimum required load described by the maximum allowed shedding fraction as imposed by the following constraint:

(15) $$\Delta u_{h,ts,p}\leq u_{ts,p}P_{h,ts}SF_{ts}$$

where $SF_{t_{s}}$ represents the assumed potential shedding fraction of each shedding technology. The profile is flat for technologies without specific sectoral dynamics.

### Conservative flexibility

The last element presented here consists of the formulation used for technologies that allow for deviations in the reference profile without compromising the technology output and with or without paying an efficiency penalty. We call these options conservative flexibility, as all the up or down flexibility must eventually be recovered with an action in the opposite direction. Some examples of these technologies are residential and service appliances such as dishwashers, washing machines, fridges or freezers [4,41]; electric heating appliances with active or passive storage [42], [43], [44]; electric vehicles with smart charging or vehicle-to-grid enhancements [45]; industrial processes with opportunities for flexible programming of their operations [4,[46], [47], [48]]; and various kinds of batteries and storage technologies [[49], [50], [51],^51^].

To model such a vast group of technologies, they were grouped into 4 different archetypes: load shifting for typical demand response and active thermal storage; smart charging of electric vehicles; vehicle-to-grid; and storage technologies. Each of these groups is represented under a specific formulation in the model and can be applied to all technologies considered under each category. However, all formulations share three elements in common: a balance constraint, a capacity constraint, and a saturation constraint, and each of the elements is interpreted differently for each archetype. It is important here to mention that these 4 archetypes refer only to the fundamental constraints ruling the behaviour of the different conservative flexible technologies; however, the technologies in the model are explicitly included (i.e., each flexible technology is independently accounted for in the model).

The energy balance states that the net energy demand should remain constant for the considered time window, and the use of time windows is adopted to maintain a linear formulation of the balance. This implies that the net balance of the upwards and downwards gross shifted load within the time window should be equal to the corresponding losses, if any, as follows:

(16) $$\sum_{h\;\in \;TW_{tf}}\Delta {q^{up}}_{h,tf,p}+\sum_{h\;\in \;TW_{tf}}\Delta {q^{dw}}_{h,tf,p}=\sum_{h\;\in \;TW_{tf}}l_{h,tf,p}$$

Both upward and downward shifts are subject to a physical capacity constraint determining the minimum and maximum boundaries of the feasible rescheduling capacity. For instance, this constraint in flexible heat pumps sets the maximum available upward shift equal to the difference between the reference profile and the heat pump's maximum capacity. These limits can be asymmetrical to each other and can be hourly variables. This second element is illustrated in the two following equations:

(17) $$\Delta {q^{up}}_{h,tfp}\leq \Delta {q^{max}}_{h,tf,p}$$

(18) $$\Delta {q^{dw}}_{h,tf,p}\geq \Delta {q^{min}}_{h,tf,p}$$

Finally, a saturation constraint ensures that the shifted volume does not violate a feasible operational limit, such as the storage capacity of an active storage unit or a latent heat requirement of a built environment system. These saturation limits can be either fixed or represented by a combination of parameters and variables depending on the archetype involved; therefore, the third type of constraint follows the structure below:

(19) $${v^{min}}_{h,tf,p}\leq \sum_{h\;\in \;TW_{tf}}\left[B^{up}\Delta {q^{up}}_{h,tf,p}+B^{dw}\Delta {q^{dw}}_{h,tf,p}\right]\leq {v^{max}}_{h,tf,p}$$

$B^{up}$ and $B^{dw}$ are two conceptual binary parameters used to illustrate that the saturation constraint can be imposed independently on both shift directions.

The interpretation of these three forms of constraints is presented below for all 4 presented archetypes.

#### Demand Response

This form of flexibility assumes that the installed capacity of the technology caps the application of flexibility. This directly affects the capacity constraint interpretation, stating that the maximum upward deviation available is given by the difference between the installed capacity and the use of the technology determined by the hourly profile in the following way:

(20) $$\Delta {q^{up}}_{h,tf,p}\leq \left(s_{tf,p}FC_{tf}-u_{tf,p}P_{h,tf}\right)AE_{tf,a}$$

and the maximum upward deviation is given by the ability of the technology to decrease its hourly consumption given by

(21) $$\Delta {q^{dw}}_{h,tf,p}\leq \left(1-NN_{tf}\right)u_{tf,p}P_{h,tf}AE_{tf,a}$$

The volume constraint ensures that the reallocated energy consumption within a time window does not exceed the original total consumption of the time window, upwards or downwards, as shown below.

(22) $$\sum_{h\;\in \;TW_{tf}}\Delta q_{h,tf,p}\;\leq \sum_{h\;\in \;TW_{tf}}u_{tf,p}P_{h,tf}AE_{tf,a}$$

#### Storage

The (dis)charging capacity gives the interpretation of the capacity constraint for storage. The maximum amount of flexibility that any storage technology can provide is determined by the following constraint:

(23) $$\Delta q_{h,tf,p}\leq s_{tf,p}CC_{tf}$$

The interpretation of the volume constraint for storage is marked by the storage capacity as described by the theoretical charging time of a battery according to the following constraint.

(24) $$\sum_{i\leq h}\Delta q_{i,tf,p}\;\leq s_{tf,p}CC_{tf}CT_{tf}$$

#### Smart charging and vehicle‐to‐grid

The main characteristic of these forms of flexibility is that they are dependent on the number of vehicles connected to the grid at a given moment. Thus, the upward capacity is capped by the difference between the charging capacity of connected EVs and the reference charging profile as given by:

(25) $$\Delta {q^{up}}_{h,tf,p}\leq CC_{tf}\left(s_{tf,p}-\frac{u_{tf,p}VU_{h,tf}}{AS_{tf}}\right)-u_{tf,p}P_{h,tf}AE_{tf,a}$$

The downwards flexibility is constrained by the reference consumption 7 and the non-negotiable load for smart charging:

(26) $$\Delta {q^{dw}}_{h,tf,p}\leq \left(1-NN_{tf}\right)u_{tf,p}P_{h,tf}AE_{tf,a}$$

By the discharging capacity of connected vehicles for vehicle-to-grid flexibility:

(27) $$\Delta {q^{dw}}_{h,tf,p}\leq DC_{tf}\left(s_{tf,p}-\frac{u_{tf,p}VU_{h,tf}}{AS_{tf}}\right)$$

The volume constraint for both smart charging and V-to-G is given similarly to storage, where the cumulative application of flexibility cannot exceed the difference between the available storage capacity of connected vehicles and the minimum required stored energy for the journeys of the vehicles departing in that hour given by:

(28) $$\sum_{i\leq h}\Delta q_{i,tf,p}\;\leq CC_{tf}CT_{tf}\left(s_{tf,p}-\frac{u_{tf,p}VU_{h,tf}}{AS_{tf}}\right)-\sum_{h\leq i\leq h+AJ}u_{tf,p}P_{i,tf}AE_{tf,a}$$

## Operation of gaseous networks

Integrated electricity and gas models usually focus on designing a proper nodal representation of the network based on pressure tolerances and Bernoulli equations, intending to provide detailed planning and operation optimisation [52]. Because of the large scope of the problem and specific goals of the methodology, IEM often ignores any detailed description of the gas system. However, because we aim to address seasonality, buffer opportunities, and infrastructure costs, IESA-Opt includes a simplified representation of gaseous network operation based on a daily balance dispatch approach [53]. This representation is presented below.

Gas networks, as transporters of a compressible fluid, are inherently provided with a buffer that allows for damping (i.e., the temporal discoordination between the input and output flows to the gas network) [53]. However, the operation of the network must occur within safety pressure boundaries, meaning that the size of the buffer has limits (and regions), thus requiring intraday balancing actions to keep networks functional^8^. There is no specific balancing period in this scheme. The imbalances are corrected when the magnitude of the imbalance reaches a certain predefined level [54].

A daily balancing approach was selected for activities distributed by the network of gaseous pipelines. This approach was selected first due to the previously described damping characteristic and second due to a typical daily flat price profile resulting from models with the hourly balancing of gas dispatch [55]. Such modelling choice allows for dispatching national wells and imports, considering the daily operation of the buffers (e.g., gas storage chambers), and describing other generation processes with particular sectoral dynamics such as fermentation, (bio)gasification, and methanation^9^. However, this representation cannot provide network planning or operation of circulating compressors. Finally, the same approach is used for all the gas transported in pipelines: natural gas (HD, MD, and LD), hydrogen (HD and LD), and sequestered carbon dioxide for CCUS.

Similar to the electric balancing description, the gas dispatch is described for each day accordingly with:

(29) $$u_{d,td,p}AB_{td,a,p}=u_{tp,p}P_{d,tp}AB_{tp,a,p}+\left(\Delta {q^{up}}_{d,tg,p}+\Delta {q^{dw}}_{d,tg,p}\right)AG_{tg,a}$$

Additionally, the daily dispatch technologies, analogous to the power dispatch, are bounded by their daily availability profiles and installed capacities accordingly with:

(30) $$u_{d,td,p}\leq s_{td,p}\Gamma_{td}P_{d,td}$$

## Networks’ infrastructure description

The infrastructure of the networks imposes a limitation on the system in terms of the extent to which an activity can be carried out within a certain time frame and geographical area. This restriction provides an extra incentive for flexibility, as it can avoid network reinforcement costs [52]. Furthermore, these infrastructure descriptions help to better represent the expected transitional costs, as the energy system must adapt to enable the deployment of infrastructure-intensive technologies, such as CCUS, hydrogen, and district heating. The infrastructure representation adopted in IESA-Opt is presented in Table 1.

**Table 1:** Considered infrastructure technologies in IESA-Opt.

Technology	Activity	Time frame
Final natural gas HD grid pipeline	HD Final natural gas	1 day
Final natural gas MD grid pipeline	MD Final natural gas	1 day
Final natural gas LD grid pipeline	LD Final natural gas	1 day
Hydrogen HD grid pipeline	HD Hydrogen	1 day
Hydrogen LD grid pipeline	LD Hydrogen	1 day
CCUS grid pipeline	CCUS	1 day
HV Electricity grid cable	HV Electricity	1 hour
MV Electricity grid cable	MV Electricity	1 hour
LV Electricity grid cable	LV Electricity	1 hour
LT Heat distribution network pipeline	LT Heat distribution network	1 hour

As shown in Table 1, the activities constrained by available infrastructure are described with daily and hourly timeframes. For the hourly ones, infrastructure limits the volumes of the activity in a time frame accordingly with:

(31) $$\begin{array}{ccc} & & \left(u_{t,p}P_{h,t}+\Delta u_{h,ts,p}\right)AB_{t,a,p}+\left(\Delta {q^{up}}_{h,tf,p}+\Delta {q^{dw}}_{h,tf|tf\;\neq \;tf_{b},p}\right)AE_{tf,a}\leq s_{ti_{h},p}\Gamma_{ti_{h}}\\ & & \forall \;a\;\left|\;a\in A^{e}\;\;\;\forall t\;\right|AB_{t,a,p}>0 \end{array}$$

Similarly, the model considers the following constraint for the daily described infrastructure technologies, ${t_{i}}_{d}$:

(32) $$\begin{array}{ccc} & & \left(u_{tp,p}P_{d,tp}+\Delta u_{h,tc,p}+\Delta u_{h,ts,p}\right)AB_{tp,a,p}+\left(\Delta {q^{up}}_{d,tf,p}\right)AG_{tf,a}\leq s_{ti_{d},p}\Gamma_{ti_{d}}\\ & & \forall a\left|a\in A^{g}\;\forall \;t\right|AB_{t,a,p}>0 \end{array}$$

Other elements of the energy infrastructure, such as transformers and buffers, are considered operational technologies. Thus, this formulation does not represent these technologies as it only refers to infrastructure that exerts no action other than enabling the flow of an activity to a certain volume.

## Declaration of Competing Interests

The authors declare that they have no known competing financial interests or personal relationships that could have influenced the work reported in this paper.

## Figures and Tables

**Table 2:** Summary of the inventory of emission sources and forms in the Netherlands. LULUCF.

Source	Activity detailed	Form	Units	1990		2016	2017	Modelled
Energy-related	Fuel Combustion	CO2	MtonCO2eq		154.5	158.6	156.2	Explicitly
Agriculture	Enteric fermentation	CH4	MtonCO2eq		9.2	8.8	8.7	MACC
Agriculture	Manure management	CH4	MtonCO2eq		5.4	3.9	3.9	MACC
Industrial Production	Ammonia production	CO2	MtonCO2eq		3.7	3.8	3.9	Explicitly
Waste	Managed waste disposal on land	CH4	MtonCO2eq		13.7	2.8	2.6	Aggregated
Energy-related	Fuel Combustion	CH4	MtonCO2eq		0.9	1.6	1.7	Explicitly
Agriculture	Inorganic fertilisers	N2O	MtonCO2eq		2.5	1.5	1.6	MACC
Industrial Production	Refrigeration	HFC	MtonCO2eq		0	1.5	1.5	MACC
Agriculture	Organic N fertilisers	N2O	MtonCO2eq		0.8	1.3	1.4	MACC
Energy-related	Fugitive Emissions	CO2	MtonCO2eq		0.9	1.1	1.1	Excluded
Agriculture	Urine and dung from grazing animals	N2O	MtonCO2eq		3	0.9	0.9	Aggregated
Agriculture	Manure management	N2O	MtonCO2eq		0.9	0.8	0.8	MACC
Industrial Production	Caprolactam production	N2O	MtonCO2eq		0.7	0.8	0.8	Explicitly
Industrial Production	Other mineral use	CO2	MtonCO2eq		0.48	0.77	0.79	Aggregated
Agriculture	Cultivation of organic soils	N2O	MtonCO2eq		0.9	0.7	0.7	Aggregated
Industrial Production	Other chemical industry	CO2	MtonCO2eq		0.6	0.5	0.7	Explicitly
Agriculture	Indirect N2O Emissions from managed soils	N2O	MtonCO2eq		1.6	0.6	0.6	Aggregated
Energy-related	Fuel Combustion	N2O	MtonCO2eq		0.3	0.6	0.6	Explicitly
Energy-related	Fugitive Emissions	CH4	MtonCO2eq		1.9	0.6	0.5	Excluded
Industrial Production	Petrochemical and carbon black production	CO2	MtonCO2eq		0.3	0.5	0.5	Explicitly
Industrial Production	Indirect CO2 emissions	CO2	MtonCO2eq		0.9	0.5	0.5	Aggregated
Agriculture	Crop residues	N2O	MtonCO2eq		0.5	0.3	0.3	Aggregated
Industrial Production	Cement production	CO2	MtonCO2eq		0.42	0.24	0.3	Aggregated
Industrial Production	Nitric Acid production	N2O	MtonCO2eq		6.1	0.3	0.3	Aggregated
Industrial Production	Petrochemical and carbon black production	CH4	MtonCO2eq		0.3	0.3	0.3	Explicitly
Industrial Production	Lime production	CO2	MtonCO2eq		0.16	0.17	0.23	Aggregated
Industrial Production	Paraffin wax use	CO2	MtonCO2eq		0.1	0.2	0.2	Aggregated
Industrial Production	Other ODS Substitute	HFC	MtonCO2eq		0	0.2	0.2	Aggregated
Waste	Wastewater treatment and discharge	CH4	MtonCO2eq		0.3	0.2	0.2	Excluded
Industrial Production	Ceramics	CO2	MtonCO2eq		0.14	0.12	0.12	Aggregated
Industrial Production	Other Soda Ash uses	CO2	MtonCO2eq		0.07	0.12	0.12	Aggregated
Industrial Production	Fluorochemical production	HFC	MtonCO2eq		6.4	0.2	0.1	Aggregated
Industrial Production	Lubricant use	CO2	MtonCO2eq		0.1	0.1	0.1	Aggregated
Industrial Production	SF6 and PFC from other products use	SF6	MtonCO2eq		0.3	0.1	0.1	Aggregated
Industrial Production	N2O from product uses	N2O	MtonCO2eq		0.2	0.1	0.1	Aggregated
Waste	Biological treatment of solid waste	CH4	MtonCO2eq		0	0.1	0.1	Excluded
Waste	Biological treatment of solid waste	N2O	MtonCO2eq		0	0.1	0.1	Excluded
Waste	Wastewater treatment and discharge	N2O	MtonCO2eq		0.2	0.1	0.1	Excluded
Industrial Production	Glass production	CO2	MtonCO2eq		0.14	0.1	0.008	Aggregated
Agriculture	Liming	CO2	MtonCO2eq		0.2	0	0	Excluded
Industrial Production	Fluorochemical production	PFC	MtonCO2eq		0	0	0	Excluded
Industrial Production	Iron and steel production	CO2	MtonCO2eq		0.05	0	0	Excluded
Industrial Production	Aluminium production	CO2	MtonCO2eq		0.45	0.1	0	Excluded
Industrial Production	Aluminium production	PFC	MtonCO2eq		2.6	0	0	Excluded
Industrial Production	Other non-specified	CO2	MtonCO2eq		0	0	0	Excluded
Industrial Production	Semiconductors	PFC	MtonCO2eq		0	0.1	0	Excluded
Industrial Production	Other process emissions	CO2	MtonCO2eq		0.1	0	0	Excluded
Total					222	195	193	

**Fig. 2:**
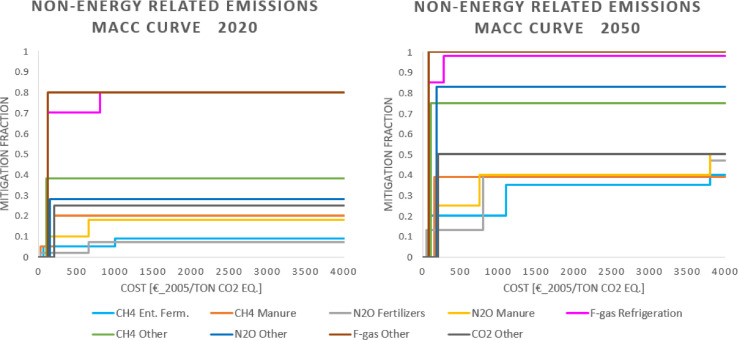
MACC curves of non-energy-related GHG emissions for 2020 and 2050 as considered in IESA-Opt^1^. ^1^Note: MACC costs reported in the figure are expressed in €_2005 as those were the units used by the data source, but input data in IESA-Opt is expressed in €_2019.

**Fig. 3:**
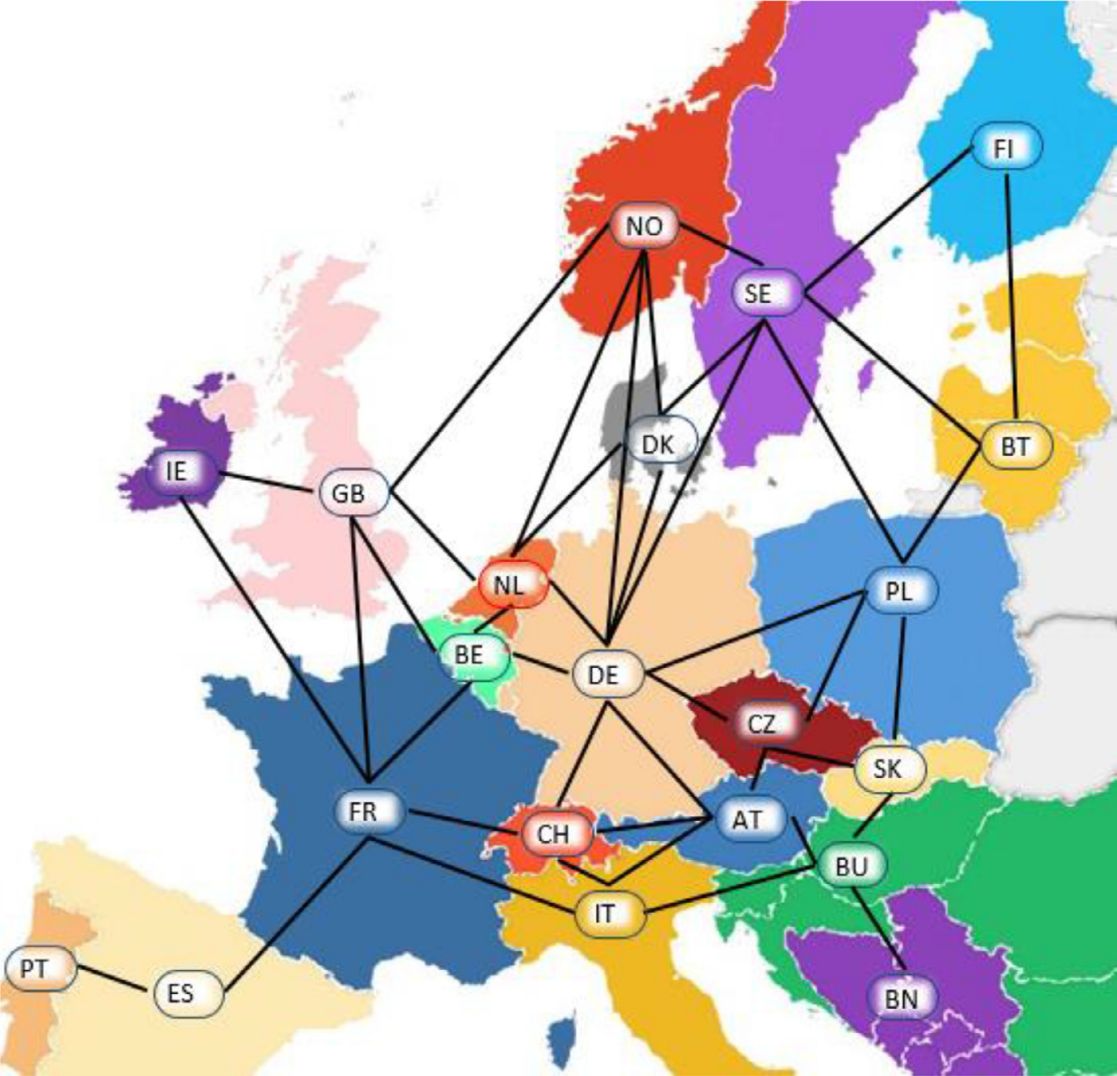
Nodal representation of the European power system considered in IESA-Opt.

**Table:** 

Technology	Investment 2020	Investment 2050	FOM	VOM	LT	Ramp	Eff.
Units	[M€/GW]	[M€/GW]	[M€/GW-y]	[M€/GWh]	[y]	[%]	[GWhf/Gwhe]
Coal old	1823.8	1809.4	18.3	2.6	40	0.5	2.41
Coal	1823.8	1809.4	18.3	2.3	40	0.5	1.79
CCGT old	899.2	892.1	11.3	1.8	30	0.8	2.49
CCGT	899.2	892.1	11.3	1.6	30	0.9	1.69
Gas CHP	1016.0	1008.0	12.7	1.6	20	0.9	2.89
GT	562.0	557.5	7.0	1.0	20	1	2.81
Oil	613.5	613.5	7.8	2.6	20	1	3.01
Waste	2254.4	2254.4	112.7	2.6	20	1	3.13
Other RES	3576.9	3191.1	0.0	3.8	20	1	1
Biomass	2657.4	2229.1	42.3	2.6	20	1	2.44
Nuclear	5636.0	5636.0	70.5	6.4	60	0.2	3.12
Hydro	4284.0	4205.1	10.8	1.1	45	1	1
Onshore Wind	1259.7	1074.5	17.2	1.6	20	1	1
Offshore Wind	1830.8	1102.0	186.0	2.1	20	1	1
Solar	764.9	279.1	2.0	0.4	20	1	1
Pumped Hydro	1252.4	1252.4	4.8	0.0	20	1	1.43
Undispatched	NA	NA	NA	3000	NA	1	NA
Interconnection	(220 - 650)	(220 - 650)	(5.5 - 16.25)	0	50	1	1.02

## Appendix A ‐ Consideration of non energy‐related emissions in IESA‐Opt

To cover all the GHG emissions forms considered within the decarbonisation reduction targets, data from the 2017 national GHG emission inventory report were used [56]. This helped identify which emissions were not yet covered by the model and prioritise which emission sources should be included to increase robustness in the emission reduction analysis. A summary of the sources, emission activity, emission form, evolution from 1990 to 2017, and how the model deals with each is presented in Table 2.

Based on the inventory shown in the above table, the following approach included the emission sources in IESA-Opt. First, from all the emissions that were not yet explicitly accounted for by the activities in IESA-Opt as fuels or industrial processes (which accounted for 85% of the total emissions in 2017), the most significant ones were extracted—being the latter: enteric fermentation (CH4), manure management (CH_4_ and N_2_O), organic and inorganic fertilisers (N_2_O), and refrigeration (HFC). Another reason for selecting these sources is that reliable data were found to incorporate their MACC curves into the model accordingly with the IMAGE model database [22,57].

Based on the above data, the following activities were defined to include all non energy-related emissions in IESA-Opt:

1 CH_4_ emissions from enteric fermentation.
2 CH_4_ emissions from manure management.
3 CH_4_ emissions from other sources (aggregated).
4 N_2_O emissions from manure management.
5 N_2_O emissions from fertiliser utilisation.
6 N_2_O emissions from other sources (aggregated).
7 F-gas emissions from the use of HFC as a refrigeration fluid.
8 F-gas emissions from other sources (aggregated).
9 CO_2_ emissions from other sources (aggregated).

The resulting MACC curves used in IESA-Opt for the nine abovementioned sources of non energy-related GHG emissions are reported in Figure 2.

## Appendix B ‐ EU Power system representation in IESA‐Opt

The representation of the EU power system is mainly extracted from COMPETES model [27] in terms of nodal representation and technologies considered, as well as the parameters used for IESA-Opt. In terms of the nodal representation, only one modification was made to COMPETE's representation, and this was to join both eastern and western Denmark nodes into one single node. The complete nodal representation is shown in Figure 3.

The operational parameters of the generation technologies required by the model consist of both economical and operational components. The list of technologies, and operational parameters assumed for the European power system are shown in the following table^10^.

## Footnotes

1 Approximate the (residual) load duration curve by dividing a year into a limited number of time slices (typically 4-12) to represent seasonal, daily and diurnal variations in demand and supply. [58]

2 That literature review is used as the pillar of the foundation of the model whose methodology is presented in this article, so it is recommended to revise it if further information is required.

3 A model run where all the model capabilities are enabled solves optimally in less than 8 hours. It uses Gurobi 9.0’s barrier method in an i7 processor with 6 cores, a RAM memory of 32 GB and a SSD enabled to share memory capacity for processing. The problem size is approximately 20 million variables, 25 million constraints, and 150 million non-zeros, resulting in 90 GB of maximum memory use.

4 The model can represent any activity or energy carrier for different geographical scales. This is ideal for modelling regions, municipalities or clusters. However, this framework it is not practical for spatial oriented aspects or locational planning decisions, as there are better spatial-based solutions to deal with these types of problems [12].

5 The first term represents the variable costs due to the use of the technologies; the second one, the investment costs resulting from investment decisions; the third one, the non-recoverable capital costs from premature decommissioning; the fourth one, the costs of retrofitting existing technologies; and the last one the fixed operational and maintenance costs of the technological stock.

6 The profiles are normalized and extracted from historical datasets such as the wind and solar availability in the Netherlands and the other 20 considered EU regions; the load profile of the Netherlands and EU regions; reference EV charging and connection profiles; temperature profiles; and a flat profile. Due to availability of data, thus far only 84 hourly profiles have been included, but every technology is assigned to one of them, which means that many technologies share profiles. However, if more data becomes available the model is already enhanced to easily include it into the database, and would not result in increased computational times.

7 The EVs reference consumption is an input data that can easily be changed to explore different scenarios. Currently, the reference charging profile is based on the standard pattern in which EV users connect their vehicles to charge right after their journey, resulting in the characteristic “two-spike” profile. Similarly, the EV's usage profile is also provided as input data.

8 There are different types of balancing actions designed accordingly with the size of the imbalance. As reference of the magnitude, no balancing action is required for hourly imbalances of ∼2% of the daily market volume. In average, 3 balancing actions per day were required between November 5^th^ 2019 and December 4^th^ 2019 [53] (high demand season).

9 Methanation, as an electricity consumer, is already subject to hourly shedding constraints (section 6.2). Thus, the daily gas dispatch formulation further restricts its operation.
